# Postoperative Vision Loss Following Bariatric Surgery: Case Report and Literature Review

**DOI:** 10.7759/cureus.87995

**Published:** 2025-07-15

**Authors:** Jawdat Alali, Umm E Amara, Mahmoud Tabouni, Umm e Nashrah, Nissar Shaikh

**Affiliations:** 1 Critical Care, Hamad Medical Corporation, Doha, QAT; 2 Critical Care, Deccan College of Medical Sciences, Hyderabad, IND; 3 Internal Medicine, Hamad Medical Corporation, Doha, QAT; 4 Surgical Intensive Care, Hamad Medical Corporation, Doha, QAT

**Keywords:** blindness, hypertension, metabolic surgery, obesity, pres, seizures, vision

## Abstract

Postoperative blindness following non-ophthalmic surgeries is a rare but devastating complication, most commonly associated with spine and cardiovascular procedures. Thus far, there have been no reports of total blindness after metabolic surgeries in the literature.

We present a case of transient total vision loss after laparoscopic sleeve gastrectomy in a young obese female patient with poorly controlled type 2 diabetes mellitus and hypertension on antihypertensive agent. Following induction of general anesthesia, the patient experienced severe hypertensive episodes lasting approximately five minutes, although the remainder of the intraoperative course was uneventful. She was transferred to the surgical intensive care unit (SICU) for postoperative monitoring. 30 minutes later, she reported sudden complete blindness while remaining hemodynamically stable and alert. During transfer for neuroimaging, she developed a generalized seizure and a decreased Glasgow Coma Scale (GCS) score, necessitating endotracheal intubation and mechanical ventilation. Brain CT and MRI findings were consistent with posterior reversible encephalopathy syndrome (PRES).

Although her GCS improved within 24 hours, visual impairment persisted until gradual recovery commenced on the second postoperative day, with full restoration by day three. The patient was subsequently transferred to the ward and later discharged with close outpatient follow-up. This case highlights PRES as a rare cause of perioperative vision loss and underscores the critical importance of rigorous blood pressure management in the perioperative period to mitigate neurological complications.

## Introduction

Visual loss following anesthesia and surgery for non-ocular indications is an uncommon but potentially devastating complication. A review of postoperative visual field deficits after non-ophthalmic procedures identified spinal surgeries and cardiothoracic bypass operations as among the highest-risk interventions. The predominant etiologies of blindness in these cases include ischemic optic neuropathy, accounting for approximately 81%, and central retinal artery occlusion; both conditions typically result in irreversible visual impairment [1].

Crosby et al. reported a case of reversible blindness with complete visual recovery occurring in the setting of preeclamptic toxemia and hemolysis, elevated liver enzymes, and low platelets (HELLP) syndrome, associated with posterior reversible encephalopathy syndrome (PRES) [2].

The prevalence of metabolic surgeries is rising exponentially due to their significant role in the improvement, prevention, or resolution of various metabolic comorbidities, including obesity, type 2 diabetes mellitus, hypertension, cardiovascular disease, and obstructive sleep apnea. The overall incidence of major complications following metabolic surgery is approximately 4% [3,4].

PRES is a neurotoxic clinical-radiological syndrome characterized by symptoms such as altered mental status, headache, seizures, and visual disturbances. It is frequently precipitated by acute hypertension and failure of cerebral autoregulation, predominantly affecting the posterior circulation. Diagnosis is supported by MRI demonstrating bilateral, symmetrical hyperintensities on T2-weighted and fluid-attenuated inversion recovery (FLAIR) sequences, typically involving the parietal and occipital lobes. The diagnosis of PRES is established when these clinical and radiographic features are present in the absence of alternative etiologies such as cerebral infarction, infection, or demyelinating disease [5].

An increasing number of case reports have documented PRES presenting with transient total vision loss, followed by complete visual recovery, particularly in the context of hypertensive disorders. We present a case of transient total blindness occurring in the immediate postoperative period, with subsequent full restoration of vision.

## Case presentation

A 42-year-old female patient was admitted for laparoscopic sleeve gastrectomy with a BMI of 34.4 kg/m². Preoperative evaluation by cardiology and internal medicine optimized her chronic medical conditions, including poorly controlled type 2 diabetes mellitus and well-controlled essential hypertension. She had no documented history of seizures or epilepsy. Her regular antihypertensive medication was amlodipine 10 mg once daily, which she continued on the morning of surgery. Upon arrival in the operating room, her baseline non-invasive blood pressure (NIBP) was 140/90 mmHg.

General anesthesia was induced with propofol 180 mg and fentanyl 200 µg, and rocuronium 60 mg was used to facilitate endotracheal intubation. Anesthesia was maintained with sevoflurane (1.5-2%) in a 50:50 oxygen-air mixture and remifentanil was administered via target-controlled infusion (TCI) with an initial effect-site concentration target of 3 ng/mL. Mechanical ventilation was volume-controlled (tidal volume 6-8 mL/kg, respiratory rate 12-14/min, positive end-expiratory pressure (PEEP) 5 cm H₂O), with end-tidal carbon dioxide (EtCO₂) maintained at 35-40 mmHg and peripheral capillary oxygen saturation (SpO₂) >98%. The patient was positioned supine, and the operating table was adjusted to a reverse Trendelenburg position with a slight right lateral tilt. Approximately five minutes post-induction, the patient developed transient hypertension with NIBP peaking at 180/50 mmHg. This was attributed to a sympathetic surge during laryngoscopy. A 10 mg IV bolus of labetalol was administered, followed by adjustment of the remifentanil TCI, increasing the effect-site concentration from 3 ng/mL to 4 ng/mL, alongside deepening of anesthesia with sevoflurane titration. Blood pressure normalized to 140/90 mmHg and remained stable throughout the 75-minute procedure. Mean arterial pressure (MAP) was closely monitored and maintained between 70-90 mmHg. Intraoperatively, 1.5 L of crystalloids were administered, with minimal blood loss of less than 50 mL. The patient was extubated uneventfully and transferred to the SICU awake, hemodynamically stable, and comfortable, with pain managed by fentanyl boluses and paracetamol.

Approximately 30 minutes later, the patient was observed to have upward deviation of the eyeballs and tongue biting, suggestive of seizure activity. Initially, this episode was attributed to a possible fentanyl-related dystonic reaction; however, the patient remained fully conscious and responsive to verbal commands throughout, raising suspicion for a partial seizure.

A few minutes later, the patient reported complete bilateral vision loss, which was confirmed on examination to be total blindness, with visual perception limited to hand movements and light only. Pupils were equal in size and reactive to light. She remained hemodynamically stable and continued to follow commands by moving all extremities.

**Figure 1:**
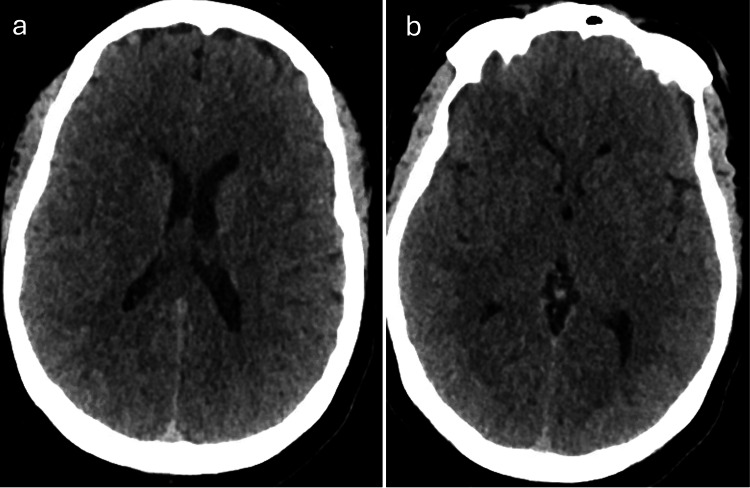
Axial non-contrast brain CT scans showing no detectable abnormalities in the parietal lobe (a) and occipital lobe (b)

**Figure 2:**
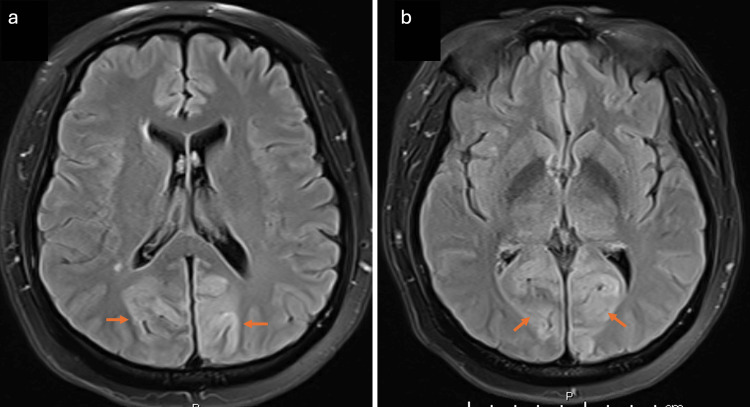
MRI of the brain. FLAIR images show areas of high signal intensity (arrows) involving the cortical and subcortical white matter regions of the bilateral parietal lobe (a) and occipital lobe (b). FLAIR: Fluid-attenuated inversion recovery

**Figure 3:**
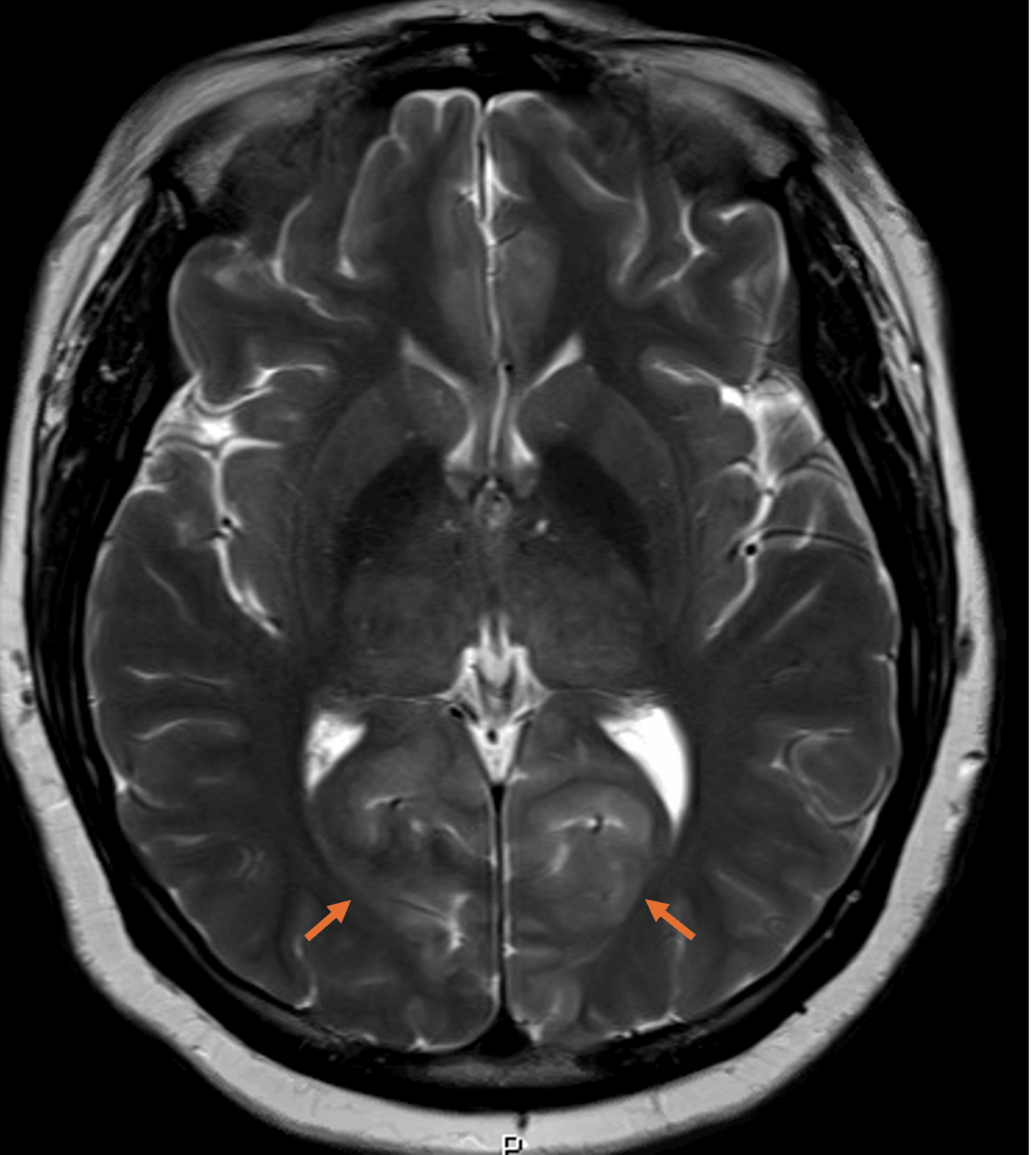
MRI of the brain T2 sequence shows corresponding occipital high signal intensity (arrows)

**Figure 4:**
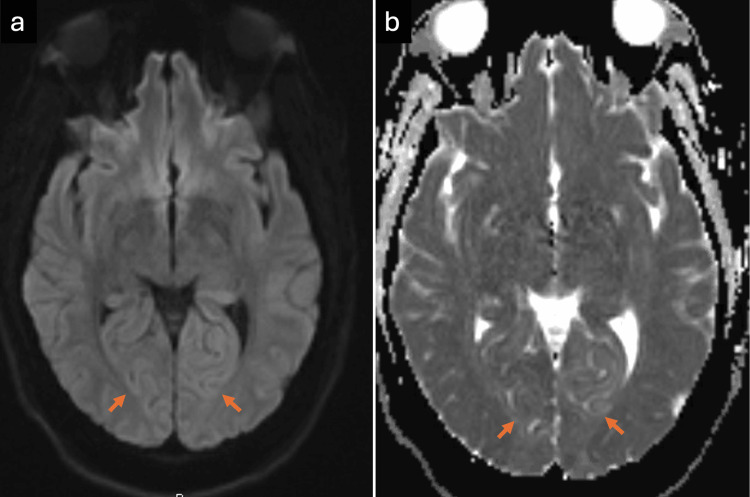
MRI of the brain (a) DWI and (b) apparent diffusion coefficient sequences show no diffusion restriction (arrows) in the corresponding occipital lobe regions, consistent with vasogenic edema. DWI: Diffusion-weighted imaging

The patient underwent evaluation for acute ocular blindness to exclude primary ocular pathology, prompting an urgent non-contrast CT scan of the brain. During the imaging procedure, she experienced a generalized tonic-clonic seizure. Despite spontaneous seizure cessation, the patient remained somnolent with a depressed Glasgow Coma Scale (GCS) score, necessitating endotracheal intubation in the CT suite for airway protection. Subsequently, she was transferred to the surgical intensive care unit (SICU) while intubated and mechanically ventilated. The brain CT scan demonstrated no detectable abnormality (Figure 1). Subsequent contrast-enhanced MRI of the brain demonstrated areas of high signal intensity involving the cortical and subcortical white matter regions of the bilateral parieto-occipital lobes on FLAIR sequences (Figure 2), corresponding occipital hyperintensities on the T2-weighted sequence (Figure 3), and no diffusion restriction in the same regions on diffusion-weighted imaging and apparent diffusion coefficient maps, indicating vasogenic edema of the bilateral cerebellar cortices and increased signal intensity in the bilateral thalami (Figure 4). These imaging findings are consistent with PRES.

The patient received a weight-based loading dose of levetiracetam 4.5 grams, followed by a maintenance dose of 1 gram administered twice daily along with prophylactic dose of dalteparin 5000 units once daily. She was successfully extubated after 24 hours, remained alert and hemodynamically stable, but continued to exhibit complete blindness. By the end of day two, her vision gradually improved, and by day three, her visual function had fully recovered. She tolerated oral clear fluids and was transferred to the general ward on day five. The patient was subsequently discharged home with scheduled outpatient follow-up.

**Figure 5:**
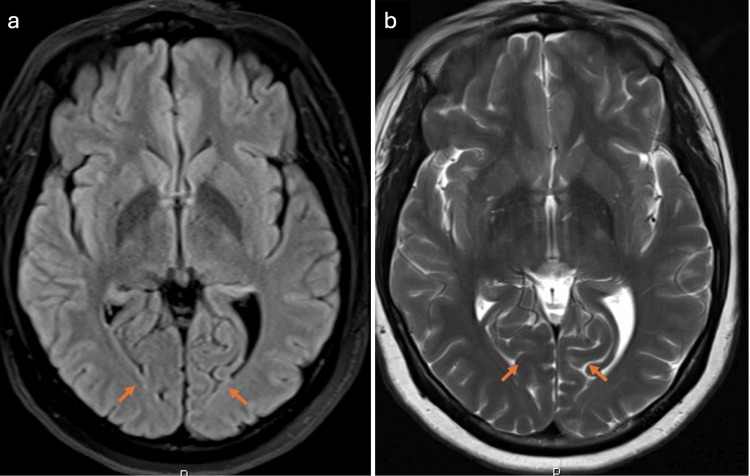
Follow-up brain MRI after three months (a) The bilateral occipito-parietal high signal intensities on FLAIR (arrows) are resolved; (b) T2-weighted imaging sequences (arrows) are resolved FLAIR: Fluid-attenuated inversion recovery

Follow-up brain MRI performed after three months demonstrated complete resolution of the previously noted bilateral occipito-parietal high signal intensities on FLAIR and T2-weighted imaging sequences (Figure 5).

## Discussion

Metabolic surgery has evolved substantially over the past seven decades, with significant advancements observed particularly in the last 15 years due to its demonstrated efficacy in controlling metabolic diseases and reducing absolute mortality risk [6]. Sleeve gastrectomy is currently the most commonly performed metabolic procedure, owing to its favorable safety profile and effectiveness [7]. The most common immediate postoperative complications following sleeve gastrectomy are hemorrhage and staple-line leaks [8].

Total blindness has not been reported as a serious immediate complication of sleeve gastrectomy or other metabolic surgeries. In our patient, episodes of severe hypertension following induction of anesthesia likely precipitated the development of total blindness, seizures, and PRES. PRES is closely associated with failure of cerebral autoregulation in the context of acute hypertensive episodes. Under physiological conditions, cerebral autoregulation maintains stable cerebral perfusion through dynamic modulation of vascular resistance. However, an abrupt elevation in blood pressure, whether from primary or secondary hypertension, can exceed the autoregulatory threshold, particularly in the posterior circulation territories where autoregulatory mechanisms are less robust. This leads to endothelial dysfunction, disruption of the blood-brain barrier, and resultant vasogenic edema, which are hallmark features of PRES [9].

Clinically, PRES manifests with acute onset of neurological symptoms including altered consciousness, visual disturbances or cortical blindness, and seizures. MRI confirms the diagnosis by demonstrating symmetrical hyperintense signals on T2-weighted and FLAIR sequences predominantly involving the parieto-occipital and posterior cerebral regions [5]. Reported etiologies of PRES encompass immunosuppression, severe infection and/or sepsis, chemotherapy, autoimmune disorders, preeclampsia, and eclampsia [10]. While PRES is more commonly reported following solid organ transplantation, primarily attributed to immunosuppressive agents like tacrolimus, it has also been documented, though rarely, after non-transplant surgical procedures such as cardiac and spine surgeries [11,12].

PRES is a major cause of morbidity and mortality in patients with eclampsia, with uncontrolled hypertension representing a frequent precipitating risk factor. Although the syndrome is termed “reversible” and typically resolves with appropriate management, complications such as generalized cerebral edema and hemiplegia can occur [13,14].

## Conclusions

While metabolic surgeries such as sleeve gastrectomy are generally considered safe, this case highlights the potential for rare but serious neurological complications like PRES. Severe hypertension following induction of anesthesia may overwhelm cerebral autoregulation, particularly in the posterior circulation, resulting in vasogenic edema and acute neurological symptoms such as seizures and reversible blindness. Careful titration of anesthetic agents is essential to prevent abrupt hemodynamic fluctuations, and perioperative blood pressure should be maintained within 20% of the patient's baseline, especially in those with chronic hypertension. Adjunct strategies, including preemptive beta-blockade and deepening anesthesia before laryngoscopy, may attenuate sympathetic surges and help stabilize intraoperative blood pressure. Early recognition of PRES through clinical vigilance and timely neuroimaging is critical, as appropriate and prompt management often leads to complete recovery. This case emphasizes the need for individualized anesthetic planning, meticulous hemodynamic monitoring, and heightened awareness of PRES risk factors to reduce the likelihood of neurological morbidity and ensure safe outcomes in routine surgical procedures.
